# Corrosion resistance and bioactivity enhancement of MAO coated Mg alloy depending on the time of hydrothermal treatment in Ca-EDTA solution

**DOI:** 10.1038/s41598-017-08242-0

**Published:** 2017-08-22

**Authors:** Seo-Young Kim, Yu-Kyoung Kim, Moon-Hee Ryu, Tae-Sung Bae, Min-Ho Lee

**Affiliations:** 1 Deptartment of Dental Biomaterials and Institute of Biodegradable material, Institute of Oral Bioscience and BK21 plus project, School of Dentistry, Chonbuk National University, 567, Baekje-daero, Deokjin-gu, Jeonju-si, Jeollabuk-do 54896 Republic of Korea; 2 Division of Biotechnology, College of Environmental & Bioresource Sciences, Chonbuk National University, 79, Gobong-ro, Iksan-si, Jeollabuk-do 54596 Republic of Korea

## Abstract

In this study, a two-step surface treatment was developed to restrain the rapid primary degradation of a biodegradable Mg alloy and to improve their biocompatibility. Micro arc oxidation (MAO) coating was performed in alkaline electrolytes such as 1.0 M NaOH with 0.1 M glycerol and 0.1 M Na_3_PO_4_. Hydrothermal treatment was performed in 0.1 M Ca-EDTA (C_10_H_12_CaN_2_Na_2_O_8_) and 0.5 M NaOH solution at 90 °C for different times (6, 12, 24, and 48 h). The film morphology and chemical properties were evaluated by XRD and FE-SEM. The electrochemical and corrosion behaviors were examined in the simulated body fluid, and cytotoxicity was assessed using MC3T3-E1 cells. After MAO coating, an oxide layer containing ${\bf{P}}{{\bf{O}}}_{4}^{3-}$ formed on the surface. During the hydrothermal treatment in Ca-EDTA solution, calcium phosphate and Mg(OH)_2_ were produced via a reaction between ${\bf{P}}{{\bf{O}}}_{4}^{3-}$ on the surface and Ca^2+^ in solution. The layer with ceramics and oxides was grown on the surface with increasing hydrothermal treatment time, and improved the surface corrosion resistance. The 24 h hydrothermal-treated group showed the lowest immersion corrosion rate and high cell viability. Therefore, this treatment was the most favorable surface modification for improving the initial corrosion resistance and bioactivity of the biodegradable Mg alloy.

## Introduction

Recently, the demand for temporary implants for bone fracture and bone loss has rapidly increased. This tendency is shown for bone scaffolds in dentistry, maxillofacial surgery, and orthopedics. Commercial medical metallic implants can cause side effects such as foreign body reactions due to the long duration of implantation, inflammations resulting from material corrosion, stress shielding effects caused by the different elastic moduli between the material and bone, stress corrosion cracking of the implant caused by repeated load, and fatigue failure. Thus, secondary surgery is required when the damaged position is completely healed after implant placement. Most patients unavoidably experience physical pain and spend large amounts of money and time^1^. Moreover, additional infection and side effects may occur after secondary surgery. In this aspect, a biodegradable metal such as magnesium and its alloys are very attractive biomaterials because they have low specific gravity, superior strength-to-weight ratio compared to other biodegradable materials, and the mechanical characteristics similar to those of natural bone. Mg has been constantly studied as a biomedical implant material (stent, pin, bone plate etc.)^2–4^. However, it has a high corrosion rate in body fluids and undergoes rapid corrosion at a primary stage. This local corrosion formed on the surface decreases mechanical strength over time. For long duration implantations, these critical factors can decrease the success rate of implants. Therefore, an approach for controlling the early corrosion rate is needed to maintain sufficiently the mechanical strength during the healing process. Many surface treatment techniques have been developed for reducing the corrosion rate and enhancing the biocompatibility of magnesium alloys by a number of surface treatment methods (micro arc oxidation^5^, vacuum evaporation coating, macromolecular^6^ and ceramic coating, composite coating^7–9^, drug deposition coating^10, 11^, etc.).

Among the methods, micro arc oxidation (MAO) can create a magnesium oxide layer with different thicknesses by varying types of electrolytes, current density, and applied voltage. This oxide layer can reduce the corrosion rate of the surface upon reaction with body fluid, and prevent the peeling off caused by implantation. Furthermore, MAO coating in an electrolyte solution containing Na_3_PO_4_ deposited phosphorous ions on the surface of the Mg alloy. This surface effectively precipitates hydroxyapatite (HA) through the reaction with Ca^2+^ and OH^−^ ions in body fluids, and the HA ultimately improves the adhesion of osteoblasts^12^. The MAO-coated layer has a porous morphology due to spark anodization at high current density. According to previous studies^13–15^, irregular pores of the MAO layer cause local corrosion, and therefore various studies have been conducted to make the surface homogenous by sealing the pores (by composite coatings, sol/gel coating, drugs, particle coating, etc.)^16–19^.

Hydrothermal treatment used for an additional surface treatment after MAO coating forms a thick film on the surface and enhances corrosion resistance^20^. Particularly, a Mg(OH)_2_ layer formed by the hydrothermal treatment in NaOH solution reduces the corrosion rate of the AZ31 magnesium alloy substrate^21^. In addition, the hydrothermal treatment in Ca-EDTA solution deposits Ca ions on the surface; this surface effectively produces HA nuclei and grows them it in the body fluid^22^.

In this study, an MAO coating was applied for controlling the early corrosion of the biodegradable magnesium alloy. To improve corrosion resistance by sealing the pores and enhance the bioactivity of the magnesium surface, hydrothermal treatment was conducted with different exposure times in Ca-EDTA solution. The corrosion characteristics and biocompatibility with the different surface treatments were assessed.

## Results

### Material characteristics

**Figure 1:**
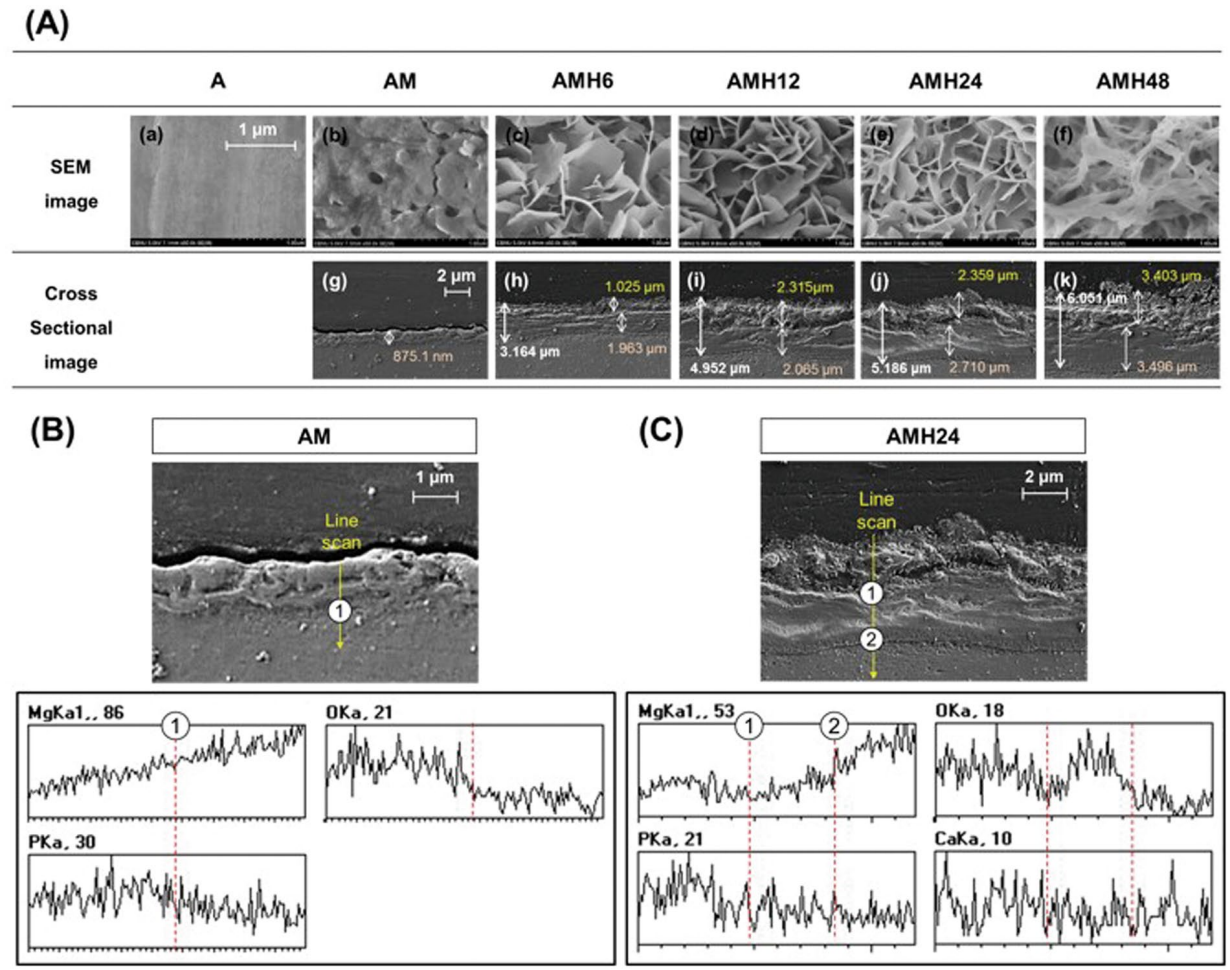
(**A**) FE-SEM images of the surface (a–f) and cross section (g–k) of specimens after A, AM, AMH6, AMH12, AMH24 and AMH48 treatments; EDS-line analysis on the cross section after (**B**) MAO coating and (**C**) MAO coating + hydrothermal treatment.

Figure 1(A) shows the surfaces and cross-sections of the specimens with different surface treatments, as observed by FE-SEM. A few pores were generated after MAO coating, giving the A group a heterogeneous surface. The thickness of the homogeneous MAO-coated layer was 880 nm. The pores produced by MAO coating were nearly completely sealed after the hydrothermal treatment, and the surface was formed with a flower-like shape. The space of the shape became denser with longer treatment. As surface treatment time increased, both the hydrothermal-treated coating layer and the initial MAO-coated layer became thicker. Unlike on the surfaces of the AMH 6, 12, and 24 groups, the surface of the AMH48 group had multiple layers of a complex network structure, and the generated coating layer showed unstable morphology.

Figure 1(B and C) and Table 1(A) show the changes in surface composition with surface treatment.

**Table 1:** Composition on the surface of the A, AM, AMH6, AMH12, AMH24 and AMH48 groups (A) before and (B) after immersion in HBSS for 14 days (at.%).

Element	A	AM	AMH6	AMH12	AMH24	AMH48
**(A)**						
Mg	96.41	59.45	48.89	45.69	43.38	30.94
Al	2.72	0.76	1.97	2.35	2.95	2.26
Zn	0.87	0.14	0.11	0.25	0.11	0.24
O		34.48	41.59	44.54	45.46	56.29
P		4.93	4.78	4.47	4.72	3.95
Na		0.24	0.27	0.25	0.7	2.55
Ca			2.39	2.45	2.68	3.77
**(B)**						
MgK	24.66	7.52	5.92	6.51	6.43	6.09
AlK	3.95	3.62	3.13	2.9	2.62	1.82
ZnL	0.95	1.5	1.8	1.19	1.3	1.29
O K	**44.91**	**43.11**	**42.96**	**41.97**	**39.69**	**40.96**
P K	**17.41**	**22.83**	**23.14**	**23.52**	**25.36**	**25.22**
NaK		0.98	0.92	0.65	0.5	0.64
Ca K	**8.12**	**20.44**	**22.13**	**23.26**	**24.1**	**24.98**

After MAO coating, the O, P, and Na ions were newly detected at the surface, and the concentration of O dramatically increased. Ca content was newly detected from the hydrothermal-treated groups (AMH groups) and increased with the increase on treatment time. Additionally, the increase in the concentration of O and Na was proportional to the hydrothermal treatment time, and the AMH48 group showed the highest increase among the AMH groups. Further, the concentration of P decreased as treatment time increased, but this didn’t show the significant difference.

**Figure 2:**
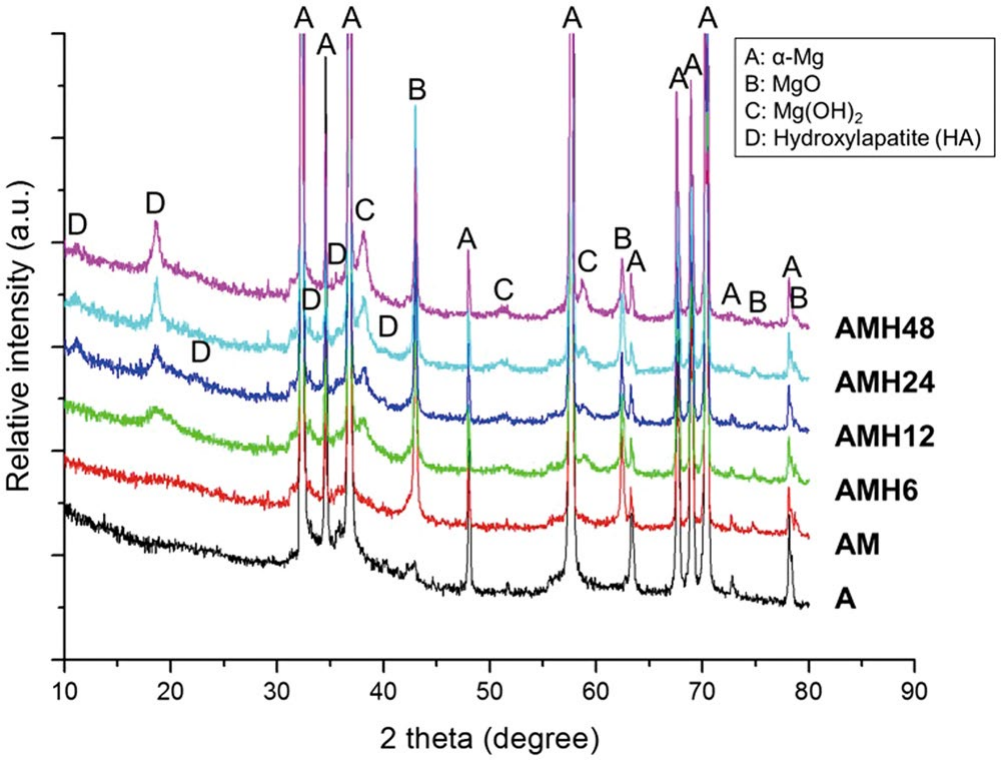
XRD patterns of the A-, AM-, AMH6-, AMH12-, AMH24- and AMH48-treated surfaces.

Figure 2 shows the results of qualitative analysis of each treated surface, using a multi-function x-ray diffractometer. After MAO coating, high MgO peaks were detected at 43.038, 62.497, 74.931, and 78.889°. After hydrothermal treatment for 6 h, many peaks were observed for Mg(OH)_2_ (at 38.269 and 50.978°) and hydroxyapatite (HA: Ca_10_(PO_4_)_6_(OH)_2_) (at 10.834, 18.829, 22.671, 32.909 and 35.475°). Their intensities increased with the treatment time. After hydrothermal treatment longer than 12 h, additional HA peak was detected at 39.815°.

### Electrochemical corrosion test

**Figure 3:**
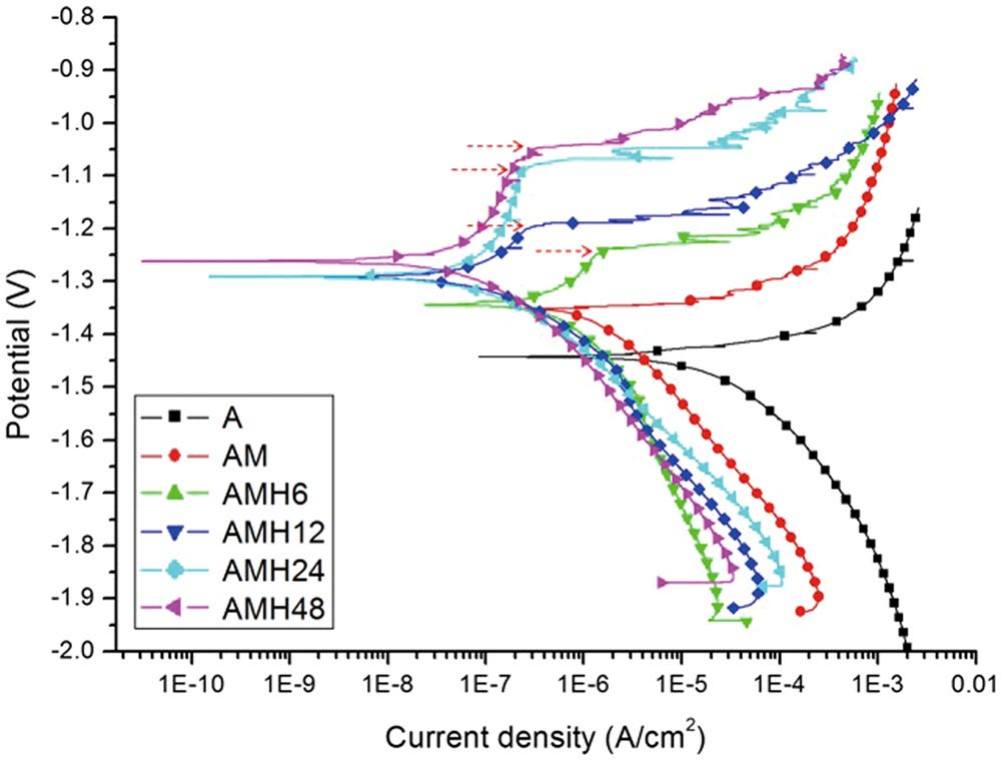
Potentiodynamic polarization curve of the A-, AM-, AMH6-, AMH12-, AMH24- and AMH48-treated surfaces.

Figure 3 and Table 2 show the results of the potentiodynamic polarization test to analyze the surface corrosion mechanism of each treated group in HBSS. After MAO coating, the current density (I_corr_) decreased to 1.66 × 10^−6^ A/cm^2^ and the corrosion potential (E_corr_) increased to −1357.39 mV. After hydrothermal treatment, it was found that the values of pitting corrosion potential (in arrows of Fig. 3) were not correlated with the treatment time. As hydrothermal treatment time increased, the current density decreased and the corrosion potential increased. In addition, a passivation area was formed after hydrothermal treatment. After hydrothermal treatment for 6 h, the current density decreased to 1.37 × 10^−6^ A/cm^2^. After hydrothermal treatment for 12 h, the current density rapidly decreased and the corrosion potential increased. In the AMH24 group, the lowest current density was shown, and the pitting corrosion potential became higher rapidly. The corrosion property of surface after the hydrothermal treatment for 48 h was similar with that for 24 h, only the corrosion potential slightly move to the novel direction.

**Table 2:** Electrochemical corrosion measurements from potentiodynamic polarization conducted in HBSS of the A-, AM-, AMH6-, AMH12-, AMH24- and AMH48-treated surfaces.

	A	AM	AMH6	AMH12	AMH24	AMH48
E_corr_ (mV)	−1439.62	−1357.39	−1351.42	−1291.78	−1292.43	−1258.64
I_corr_ (A/cm^2^)	4.11 × 10^−5^	1.66 × 10^−6^	1.37 × 10^−6^	4.69 × 10^−7^	1.95 × 10^−7^	1.86 × 10^−7^

### Immersion corrosion test

**Figure 4:**
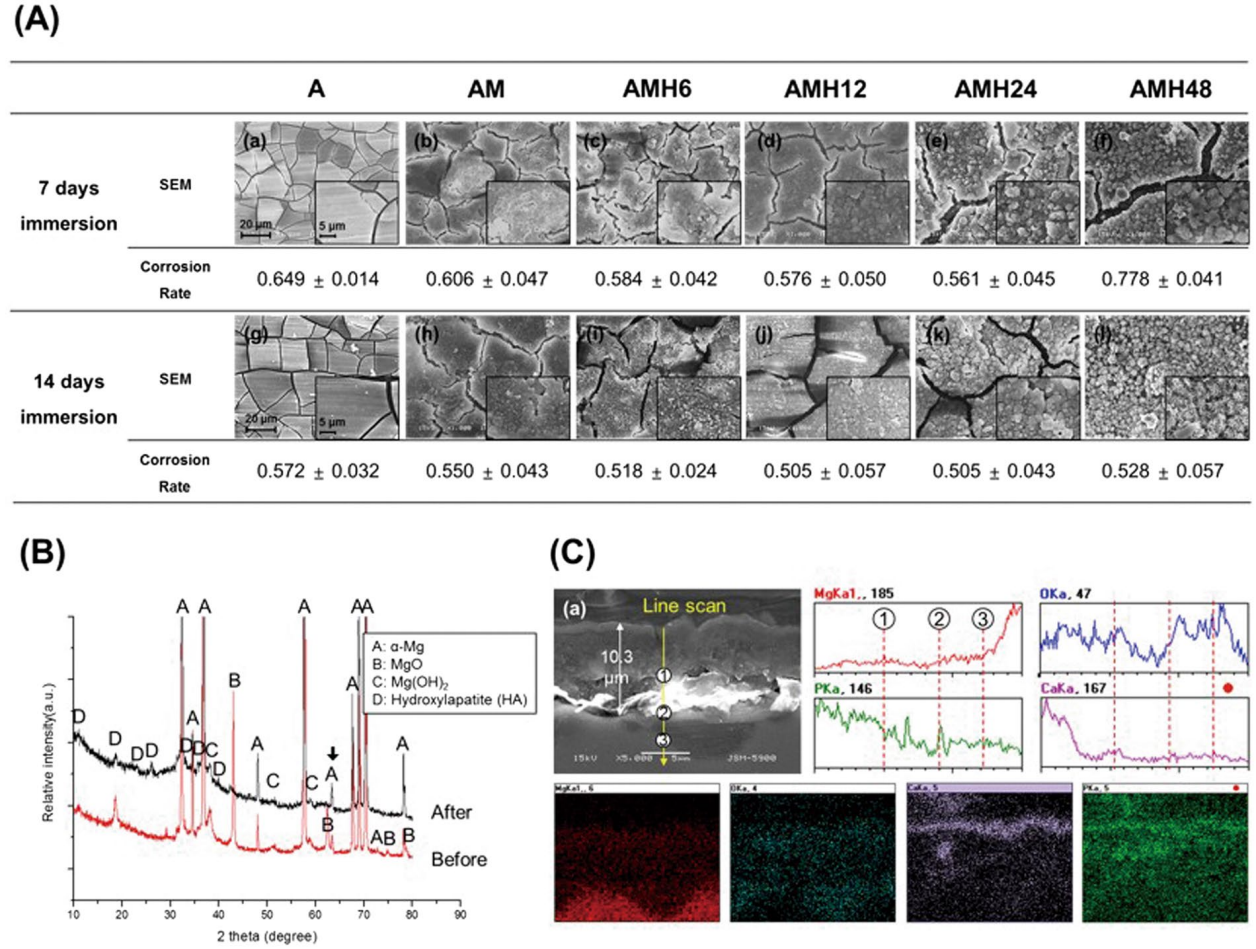
(**A**) SEM images showing surface morphologies of the A, AM, AMH6, AMH12, AMH24 and AMH48 treated surfaces; (**B**) XRD patterns on the surface of the AMH24 group before and after 14 days of immersion in HBSS; (**C**) EDS-line and -mapping analysis on the cross section of the AMH24 group after 3 days of immersion in HBSS.

As shown in Fig. 4(A), changes to surface morphologies were observed after immersing each group in HBSS. After 7 days of immersion, numerous fine cracks existed on the surface, and the space between the cracks became larger by adding each surface treatment. Moreover, precipitates with spherical morphology were observed on the surfaces of the hydrothermal treated groups over 12 h, and the amount was highest in the AMH24 and 48 groups. After 14 days of immersion, the surface cracks decreased in all groups compared to those at 7 days of immersion. The precipitates were generated on the surfaces of all hydrothermal treated groups, and the amount was proportional to the increase of treatment time.

Figure 4(A) also shows the corrosion rate calculated from the immersion test. After 7 days of immersion, the corrosion rate reduced with the addition of successive surface treatments. However, the AMH48 group showed a higher corrosion rate than the A group (untreated group). After 14 days of immersion, the corrosion rate also decreased by adding each surface treatment; the AMH12 and 24 groups showed the lowest corrosion rate. The corrosion rate of the AMH48 group was lower than that of the A and AM groups unlike after 7 days of immersion in HBSS, but it was still higher than those of the other hydrothermal-treated groups.

Table 1(B) shows the results of the EDS analysis on the surface-treated groups after soaking in HBSS. After 14 days of immersion, large amounts of O, P, and Ca were detected on the surfaces in all groups. The MAO-coated group showed higher O contents on the surface after immersion, and the concentrations of P and Ca were higher than those in the untreated group. Particularly, the concentration of Ca was markedly increased. The hydrothermal treated groups showed higher P and Ca concentrations than the groups without hydrothermal treatment, and the concentration of O became lower. Moreover, the hydrothermal-treated groups showed higher concentrations of P and Ca with the longer treatment time. In the AMH48 group, the concentrations of P and Ca were similar to those in the AMH24 group, but the concentration of O was higher.

Figure 4(B and C) shows the XRD patterns (on the surface) and the line/mapping images (on the cross section) for the AMH24 group after 14 days of immersion in HBSS. The bottom of the surface layer consisted of Mg and O, and the top of the surface layer consisted of Ca and P. A lot of new HA peaks was detected, and the intensity of MgO peaks decreased.

### *In vitro* cytotoxicity test

#### Cell proliferation test

**Figure 5:**
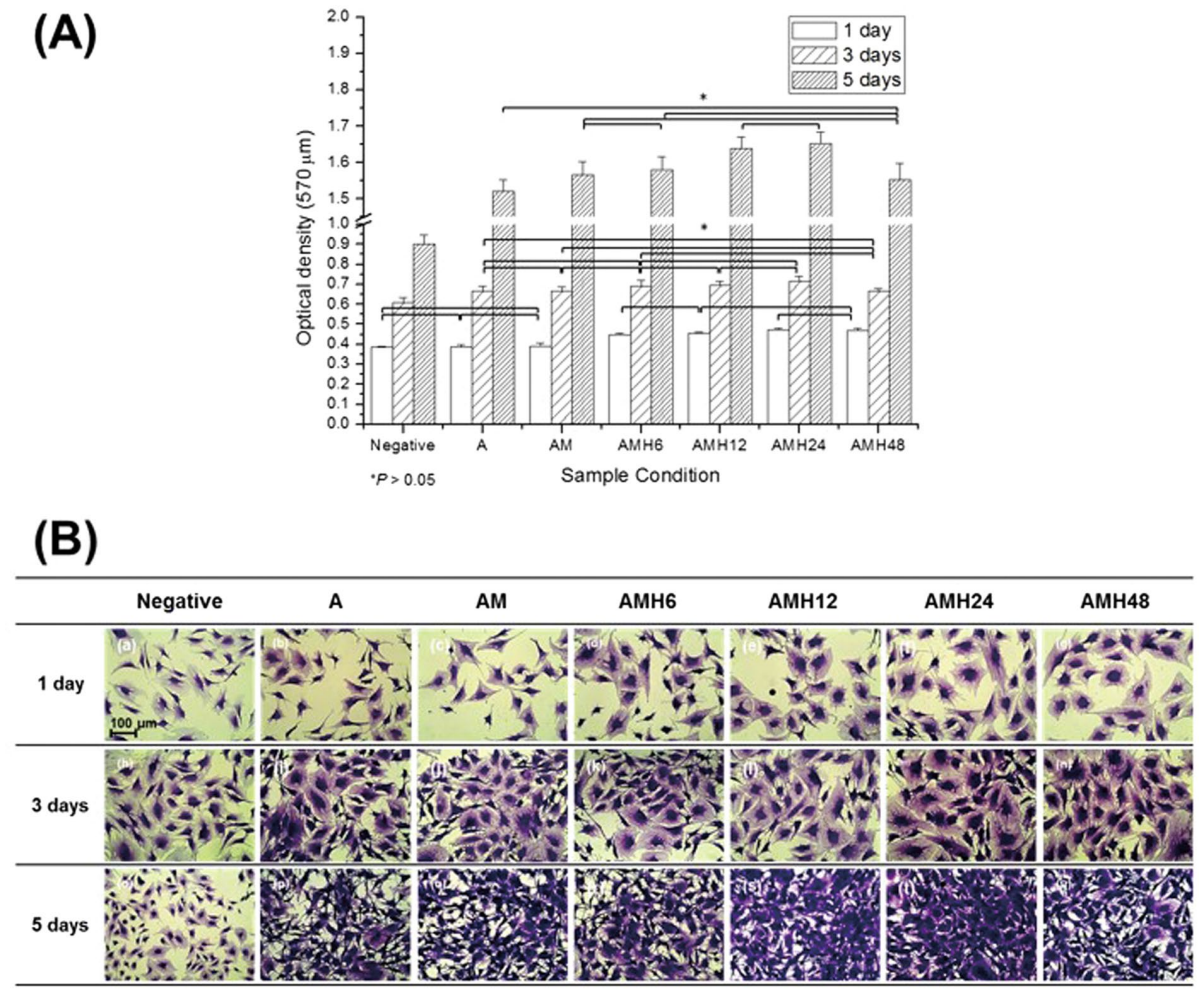
(**A**) Viability and (**B**) Morphology of MC3T3-E1 cells cultured for 1, 3 and 5 days in extracted media of the A, AM, AMH6, AMH12, AMH24 and AMH48 groups, as determined by WST assay and stain.

Figure 5(A) shows the results of the WST assay for each treated group after 1, 3, and 5 days of culture in the extracted media from each treated group, which was conducted to assess cell proliferation. The cell proliferation in each group increased based on the culturing time, and all groups showed higher values than the negative control group. After 1 day of culture, the cell viability of the hydrothermal treated groups showed a significant increase (*p* < 0.05) compared to that of the negative control group. The viability increased in proportion to the hydrothermal treatment time, but there was no significant change beyond 48 h. After 3 days of culture, the hydrothermal treated groups for ≥12 h showed the higher cell proliferation than the non-hydrothermal treated group, whereas the 48-h treated group showed lower proliferation. This trend became clearer after 5 days of culture, and the AMH 24 group showed the highest cell proliferation, with an optical density of 0.716 ± 0.026.

#### Cell morphological analysis

Figure 5(B) shows the morphologies of cells after 1, 3, and 5 days of culture in the extracted media from each treated group. The number of cells in each unit area increased in groups after hydrothermal treatment. In addition, the filopodia of the MC3T3-E1 cells became branched and extended, which means the cells maintain good adhesion with the surface of each treated group. After 1 day of culture, all groups showed similar cell viability. However, the morphological analysis for 3 days revealed that the live cells had wide and round cytoplasms in the AMH24 and 48 groups. After 5 days of culture, cell proliferation increased in the A, AM and AMH6 groups, but the filopodia of the cells in the groups became long and thin. Good cell proliferation was observed after the hydrothermal treatment for 12 h, and the cytoplasm showed the densest growth in the AMH24 group.

## Discussion

A number of studies have been performed on the clinical application of biodegradable magnesium alloys. However, the problems have been raised that the rapid corrosion rate of magnesium in the early stage may interrupt bone regeneration around the implants and delay the healing process. Therefore, in this study, we applied a composite coating of MAO and hydrothermal treatment to control the early corrosion and improve the bioactivity of a biodegradable magnesium alloy.

In agreement with previous studies^23, 24^, an uniform thick oxide layer containing fine pores was formed on the surface after MAO coating (Fig. 1(A)(b and g)). The rapid increase in the O level on the surface resulted from the production of MgO, as seen from the XRD results. In addition, ${{\rm{PO}}}_{4}^{3-}$ and Na^+^ were deposited on the surface from Na_3_PO_4_ and NaOH contained in the electrolyte used for MAO coating^12^. Previous studies have shown that heterogeneous pores on the MAO-coated layer interfere with uniform corrosion. In this study, the sealing of pores was verified using hydrothermal treatment in a 90 °C solution, and the thickness of the surface layer could be controlled by changing the hydrothermal treatment time (Fig. 1(A)(h–k)). During the hydrothermal treatment, the formation of new magnesium oxide layer (Mg(OH)_2_) was gradually promoted at the top and bottom of MAO layer after hydrothermal treatment (Fig. 4(C)). With the reaction of forming an oxide layer (MgO + H_2_O →Mg(OH)_2_, Mg + 2H_2_O → Mg(OH)_2_) in the aqueous solution, the Mg(OH)_2_ layer was produced on the surface of magnesium during the hydrothermal treatment^25^, which increased the concentration of O ions (Table 1(A) and Fig. 2). Further, ${{\rm{PO}}}_{4}^{3-}$ and Na ions contained in the MAO-coated layer bonded with Ca ions in the hydrothermal treatment solution (Ca-EDTA), so that it induced the formation of apatite. According to the XRD results, the bonded form of Ca^2+^ and ${{\rm{PO}}}_{4}^{3-}$ is HA. Ca precipitation on the surface increased with the hydrothermal treatment time, and apatite with a flower-like shape was densely formed. Through the Ca/P ratio, it can be found that Ca-deficient HA exist on surface. In contrast, the concentrations of O and Na ions largely increased in the AMH48 group. This means that the Na ion was also precipitated with Ca ions during the hydrothermal treatment over excessive time. Therefore, the surface morphology showed a multi-layer with network structure, which was similar to the surface of metallic biomaterial after alkali treatment in NaOH solution^26^.

Magnesium is easily biodegraded in the human body. The following chemical equations show the corrosion behavior of magnesium without surface treatment in SBF.

The corrosion behavior of magnesium substrate is as follows (1):

$$\begin{array}{l}(1 \mbox{-} 1)\,{{\rm{Mg}}}_{({\rm{s}})}\to {{\rm{Mg}}}_{({\rm{aq}})}^{2+}+2{{\rm{e}}}^{-}\,({\rm{anodic}}\,{\rm{reaction}})\\ (1 \mbox{-} 2)\,2{{\rm{H}}}_{2}{\rm{O}}+2{{\rm{e}}}^{-}\to {{\rm{H}}}_{2}+2{{\rm{OH}}}_{({\rm{aq}})}^{-}\,({\rm{cathodic}}\,{\rm{reaction}})\\ (1 \mbox{-} 3)\,{{\rm{Mg}}}_{({\rm{aq}})}^{2+}+2{{\rm{OH}}}_{({\rm{aq}})}^{-}\to {\rm{Mg}}{({\rm{OH}})}_{2({\rm{s}})}\,({\rm{product}}\,{\rm{formation}})\\ (1 \mbox{-} 4)\,{\rm{Mg}}{({\rm{OH}})}_{2({\rm{s}})}+2{{\rm{Cl}}}_{({\rm{aq}})}^{-}\to {\rm{Mg}}{({\rm{Cl}})}_{2}+2{{\rm{OH}}}_{({\rm{aq}})}^{-}\\ (1 \mbox{-} 5)\,10{{\rm{Ca}}}_{({\rm{aq}})}^{2+}+6{{\rm{PO}}}_{4({\rm{aq}})}^{3-}+2{{\rm{OH}}}_{({\rm{aq}})}^{-}\to {{\rm{Ca}}}_{10}{({{\rm{PO}}}_{4})}_{6}{({\rm{OH}})}_{2({\rm{s}})}\end{array}$$

In SBF, the corrosion reaction occurs between magnesium and H_2_O ((1-1) and (1-2)). A Mg(OH)_2_ film is formed on the surface (1-3). The film is transformed into soluble MgCl_2_ (1-4). Finally, the Ca_10_(PO_4_)_6_(OH)_2_(HA) is precipitated on the surface due to the consumption of Ca^2+^ and ${{\rm{PO}}}_{4}^{3-}$ (1-5). In the analysis of the surface morphology after HBSS immersion, numerous fine cracks were observed on the surface layer of the untreated group, and O, P, and Ca ions were detected. The corrosion product was formed on the surface by the oxidation pathway, and Ca_10_(PO_4_)_6_(OH)_2_ was precipitated at the same time.

These degradation and precipitation processes can be retarded or accelerated by different surface treatments. MAO coating can increase the corrosion resistance of biodegradable magnesium by changing the conditions of voltage and current density^27–29^, and control the corrosion rate in SBF^30^. Zeng^31^ and Staiger^2^ reported that surfaces modified by HA could improve the corrosion rate of magnesium alloys. Indeed, non-porous Mg(OH)_2_ oxide and calcium phosphate ceramic generated on the surface after MAO coating and hydrothermal treatment increased the corrosion potential in the electrochemical corrosion test. This agrees with the results^32^ that the MgO layer with HA and/or Mg(OH)_2_ effectively prevented the penetration of corrosive solution. The potential of metal can increase when the surface has a thin, non-porous, and insoluble protective film. After hydrothermal treatment, the excellent effect on anodic oxidation and organic corrosion inhibition were shown, and a passivation area was formed on their surface. In addition, the corrosion inhibition effect of protective oxide film on the surface became higher with the hydrothermal treatment time (Fig. 3 and Table 2).

**Figure 6:**
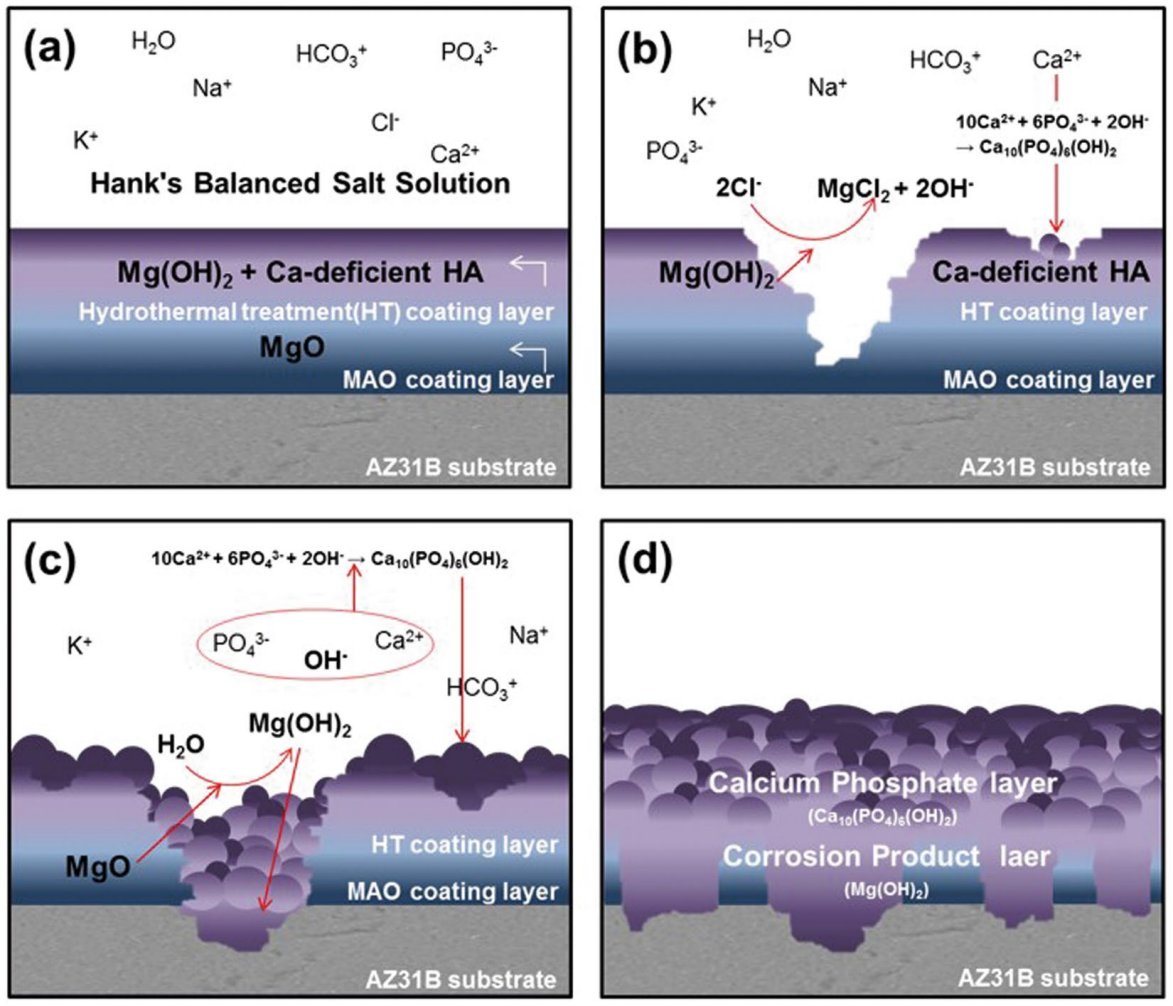
Schematic illustration (**a–d**) of the reactions on the surface of the magnesium alloy in HBSS.

The HA phases formed by the hydrothermal treatment are the most important crystal phases for biomineralization^33^, which effectively improve bioactivity of the surface by accelerating the precipitation and growth of calcium phosphate in the body^34, 35^. Some previous studies^33, 36^ explained the process of HA precipitation with the presence of Ca-deficient HA (such as dicalcium phosphate (CaHPO_4_), tricalcium phosphate [TCP: Ca_3_(PO_4_)_2_], octacalcium phosphate [OCP: Ca_8_H_2_(PO_4_)_6_.5H_2_O], etc.) in SBF containing calcium. It has been reported that Ca-deficient HA can be improve the formation of the crystallized bonelike apatite in SBF^37^. Based on the results of various studies^38–40^, the corrosion behavior of magnesium alloy with the MAO coating and hydrothermal treatment was predicted as below. The reaction is schematized in Fig. 6.

The corrosion behavior of surface-treated layer is as follows (2):

$$\begin{array}{l}(2 \mbox{-} 1)\,{\rm{Mg}}{({\rm{OH}})}_{2({\rm{s}})}+2{{\rm{Cl}}}_{({\rm{aq}})}^{-}\to {{\rm{MgCl}}}_{2}+2{{\rm{OH}}}_{({\rm{aq}})}^{-}\\ (2 \mbox{-} 2)\,10{{\rm{Ca}}}_{({\rm{aq}})}^{2+}+6{{\rm{PO}}}_{4({\rm{aq}})}^{3-}+2{{\rm{OH}}}_{({\rm{aq}})}^{-}\to {{\rm{Ca}}}_{10}{({{\rm{PO}}}_{4})}_{6}{({\rm{OH}})}_{2({\rm{s}})}\end{array}$$

In this study, through the reaction with the AMH-treated surface and HBSS, the Mg(OH)_2_ layer formed by the hydrothermal treatment starts to be transformed into soluble MgCl_2_ (2-1). The OH^−^ ionized by this process was combined with Ca^2+^ and ${{\rm{PO}}}_{4}^{3-}$ in the solution, and Ca_10_(PO_4_)_6_(OH)_2_ was formed on the surface (2-2). At the same time, Ca-deficient HA formed by the hydrothermal treatment also consistently reacted with Ca^2+^ in the solution and produced Ca_10_(PO_4_)_6_(OH)_2_, rapidly. Therefore, although the MgO layer and substrate were revealed by the localized pitting corrosion, the position was easily restored because of the fast transformation rate of Mg(OH)_2_ and deposition rate of Ca_10_(PO_4_)_6_(OH)_2_.

After 14 days of immersion in HBSS, O content on the MAO-coated layer was higher than that before immersion because of the reaction with HBSS. In addition, unlike that in the untreated group, ${{\rm{PO}}}_{4}^{3-}$ ions deposited on the surface of the AM group promoted the reactions with Ca^2+^ ions and OH^−^ ions in HBSS^12^, which led to the rapid precipitation of Ca. Furthermore, wide cracks occurred on the surface as the thick corrosion product and spherical shape of precipitate formed in the MAO-coated and hydrothermal-treated groups after immersion in HBSS. Moreover, the concentrations of P and Ca ions on the surface were high. This is because HA, which was produced on the surface of the hydrothermal-treated groups, bonded with Ca ions in HBSS solution and accelerated the precipitation of HA on the surface (Fig. 4(B)) Thus, a large amount of calcium phosphate formed on the surface with the increase in hydrothermal treatment time (particularly in groups treated for over 24 h), resulting in large increases of P and Ca ions. All hydrothermal treated groups generated a high level of calcium phosphate in HBSS and showed clear effects on bioactivity enhancement. In the hydrothermal-treated groups for ≤24 h, the corrosion rate was improved depending on the hydrothermal treatment time as predicted from the results of the electrochemical corrosion test, but the AMH48 group only showed rapid corrosion. Since the AMH48 group had more porous unstable morphology of calcium phosphate-layer than other AMH groups, chloride ion can be penetrated into calcium phosphate layer. This surface properties could promote the reaction (corrosion) between the higher amount of Mg(OH)_2_ in the surface layer of AMH48 group and Cl ions in SBF compare to that of the AMH24 group. Thus, the occurrence of MgCl_2_ became faster than the formation of calcium phosphate because the conversion of it into MgCl_2_ was accelerated by its surface. In conclusion, the electrochemical and immersion corrosion test indicated that the AMH24 group is the best surface treatment for improving corrosion resistance.

The coating layer containing the HA phase not only increases corrosion resistance but also prevents the physical detachment between the alloy surface and cells caused by gas generation and corroded debris^41^. In addition, calcium phosphate mineral (particularly HA) shows a direct correlation with bone growth and acts as an important material for orthopedic and dental implants^42^. In this study, when cytotoxicity was assessed after characteristic analysis of each surface treatment (Fig. 5), most treated groups showed higher values of cell growth than the negative control group. Furthermore, each treated group increased the number of cells per surface unit area with the culturing time, and it can be verified that the adhesion in all extracted media of each group was well maintained because the filopodia of the MC3T3-E1 cells spread such as multiple branches. These indicate that the surface treatments have low cytotoxicity. Moreover, after 1 day of cell culture, only the hydrothermal-treated groups showed significantly higher cell proliferation (*p* < 0.05) than the negative control group. The osteoclast-like multinucleated cells absorbs the apatite on the layer and contributes to bone formation by osteoblasts during the remodeling process. Thus, the osteoinductive porous implants covered by biological apatite act as natural bone in the body, and effectively promote bone generation^43^. Consequently, the thick apatite layer on the hydrothermal-treated groups induced high proliferation of osteoblast cells, which increased in proportion to treatment time. The cell proliferation was particularly high in the hydrothermal-treated group over 12 h, and the value was highest in the AMH24 group. In contrast, cell growth in the AMH48 group became lower than that in the AMH24 group. It is regarded that the increased level of pH and extracted Mg ions within the solution caused by the relatively high corrosion rate, which inhibited cell adhesion and led to lower cell proliferation.

## Conclusions

In this study, we conducted the hydrothermal treatment for different times after MAO coating to improve the corrosion resistance of a commercialized biodegradable Mg alloy and enhance its bioactivity. The hydrothermal treatment in Ca-EDTA solution after MAO coating could inhibit the surface corrosion of the magnesium alloy and improve its bioactivity in simulated body fluid, and showed lower cytotoxicity. In particular, the initial corrosion resistance of the surface was improved by increasing the hydrothermal treatment time. However, among the different tested groups, the ununiformed surface layer was formed in the surface treated for over 48 hours, and the group showed faster corrosion rate in the SBF immersion for over 7 days.

Based on all the experimental results, the AMH24 group with uniform surface layer exhibited the most effective improvement in corrosion resistance and bioactivity both early and late, and the stable cell response. Therefore, it can be suggested that the AMH24 group is the most favorable surface treatment method for application in orthopedic implants.

## Material and Methods

### Specimens & surface treatments

Square specimens (11 × 13 × 1.5 mm) were prepared from an AZ31B plate (3 wt.% Al, 1 wt.% Zn, balance Mg, KMTRA, Korea) for the analysis of material characteristics, corrosion behavior and cytotoxicity. The surface was sequentially ground from #600 to #2000 with SiC sand paper.

As the surface treatments, the hydrothermal treatment was performed at different time conditions after coating the surface of AZ31 alloy by micro arc oxidation (MAO). The groups were named as A [(A) AZ31 alloy], AM [(A) AZ31 + (M) MAO coating], AMH6, 12, 24 and 48 [(A) AZ31 + (M) MAO coating + (H) Hydrothermal treatment for 6 h, 12 h, 24 h or 48 h].

To perform MAO coating, the AZ31B specimen and a platinum plate were connected to the anode and cathode, respectively, of a DC constant-power supply (Kwangduck FA, Korea). According to a previous study^12^, the treatment was conducted in electrolyte containing 1.0 M sodium hydroxide (NaOH), 0.1 M glycerol, and 0.1 M sodium phosphate (Na_3_PO_4_) at a current density of 300 mA/cm^2^.

Hydrothermal treatment was performed by immersing the MAO-coated specimens in the mixed solution^22^ (in which pH is 13.20) of 0.1 M Ca-EDTA (C_10_H_12_CaN_2_Na_2_O_8_) and 0.5 M NaOH solution at 90 °C in a high-temperature oven (US/CC59256, A Jeon Heating Industrial Co., Korea) for the different time. Unlike previous studies^22, 32^, we chose 6, 12, 24, or 48 h as the hydrothermal treatment time for a reduction in the interval to treatment time and for diversification of species. All treated specimens were cleaned in distilled water, followed by storage in a 40 °C dryer for 24 h.

### Material characteristics

After surface treatments, the components and morphology of the surface and film were examined using a field emission scanning electron microscope (FE-SEM; SUPRA40VP, Carl Zeiss Co., Germany), with energy dispersive spectroscopy (EDS). The 15Kv of electron acceleration voltage was used for EDS analysis. The line scan and mapping analyses were performed by EDS to identify the distribution of chemical elements in coating layer. In addition, the crystal phase on the surface was analyzed using a multi-purpose high-performance x-ray diffractometer (XRD; X’pert Powder, PANalytical Co., The Netherlands) installed at the Center for University-Wide Research Facilities (CURF) at Chonbuk National University.

### Electrochemical corrosion test

The corrosion resistance of the treated groups was evaluated via the potentiodynamic polarization test. The potential voltage and current density of the groups were determined by potentiodynamic polarization scanning using an electrochemical analyzer system (PARSTAT 2273, Princeton Applied Research, USA). Ag‖AgCl/KCl(saturated), platinum, and the specimens were connected as the reference, counter, and working electrodes, respectively. Hanks’ balanced salt solution (HBSS; H2387, Sigma-Aldrich, St. Louis, MO, USA) was used as simulated body fluid (SBF) for an electrolytic solution (Table 2). The electrochemical corrosion test was carried out at a scanning rate of 3 mV/s. To determine the corrosion potential (E_corr_) and corrosion current density (I_corr_), the cathodic and anodic portions of the generated Tafel plots were fitted with linear function.

### Immersion corrosion test

The variation of Mg alloy by corrosion can be analyzed by measuring the mass loss in a solution where the media volume-to-surface area ratio is at least 50 mL/cm^2^ of HBSS^44^. Thus, each specimen was immersed in 500 mL of HBSS and kept in an incubator with 5% CO_2_ at 37 °C for 1 week and 2 weeks, respectively. After the immersion test, the specimens were removed from HBSS and dried at room temperature. The corrosion morphology and components of the surface were characterized by scanning electron microscopy (SEM; JSM-5900, JEOL, Japan), and energy dispersive spectroscopy (EDS; 7274, Oxford Instruments, England).

To measure the mass loss, the corrosion products on the surface were removed by soaking in chromic acid solution. The acid solution was manufactured by mixing 200 g/L of chromic acid, 10 g/L of silver nitrate (AgNO_3_), and 20 g/L of barium nitrate [Ba(NO_3_)_2_] according to American Society for Testing and Materials (ASTM) G1^45^. The average and standard deviation of three measurements was calculated for each group. The corrosion rate was calculated using the following equation according to ASTM G31–72^46^:

$${\rm{C}}=({\rm{KW}})/({\rm{ATD}})$$

where C is the corrosion rate (mm year^−1^, mmpy), the constant K is 8.76 × 10^4^, W is the mass loss (g), A is the specimen area exposed to solution (cm^2^), T is the time of exposure (h), and D is the density of the material (g/cm^3^).

### *In vitro* cytotoxicity test

To evaluate cell growth, the indirect cytotoxicity assessment was conducted using mouse osteoblast cells (MC3T3-E1) obtained from the American Type Culture Collection (Manassas, VA, USA). According to EN ISO 10993−12:2004^47^, the extraction was carried out by soaking each specimen in culture media for 72 h at 37 °C and 5% CO_2_. The ratio of sample area/culture media volume was 1.25 cm^2^/mL. The culture media was prepared by adding 10% fetal bovine serum (FBS, Gibco Co. USA), 500 unit/mL penicillin (Gibco Co., USA), and 500 U/mL streptomycin (Gibco Co., USA) to α-minimal essential media (α-MEM; Gibco, Carlsbad, CA, USA).

Finally, the extracted media was used in the indirect cytotoxicity assessment after diluting this media with α-MEM at a 1:2 ratio. The pre-cultured cells were seeded in 24-well plates at an initial density of 2.0 × 10^4^ cells/mL per well, and cultured for 24 h. After cell attachment, the culture media was replaced with 500 μL of the extracted media, with three wells per group. Non-extracted media was used for the control cells. Cells were cultured for 1, 3, and 5 days in an incubator that contained 5% CO_2_ at 37 °C, and the media was replaced every 24 h for cell survival. The water-soluble tetrazolium salt (WST) assay and crystal violet staining were used to assay cell viability.

#### Cell proliferation test

A cytotoxicity test was conducted using Cell Counting Kit-8 (Enzo Life Sciences Inc., NY, USA) according to the manufacturer’s instructions. The culture media was replaced with WST-8 cell proliferation reagent [2-(2-methoxy-4-nitrophenyl)-3-(4-nitrophenyl)-5-(2,4-disulfophenyl)-2H-tetrazolium, monosodium salt] for 1.5 h at 37 °C. With its higher sensitivity as a chromogenic indicator for cell viability compared with conventional tetrazolium salts, WST-8 produces a water-soluble formazan dye upon reduction in the presence of an electron carrier. The absorbance of the formazan dye was measured at 450 nm using a microplate spectrophotometer (EMax, Molecular Devices, USA).

#### Cell morphological analysis

After culturing for 1, 3, and 5 days, the culture media was removed for cell staining, and then each well was washed with phosphate-buffered saline (PBS). The cells were primarily fixed by a mixture of 0.2% glutaraldehyde (GA) and 3% formaldehyde, followed by staining with 0.3% crystal violet. The cell morphology was examined under an optical microscope (DM2500, Leica, Japan).

### Statistical analysis

Statistical analysis of the WST assay results was conducted by one-way analysis of variance. When the *p*-value was lower than 0.05, the difference between groups was considered to be statistically significant. The computer software SPSS ver12.0 (SPSS Inc., Chicago, IL, USA) was used for all statistical analyses.
